# Therapeutic Targets and Tumor Microenvironment in Colorectal Cancer

**DOI:** 10.3390/jcm10112295

**Published:** 2021-05-25

**Authors:** Gaetano Gallo, Giuseppina Vescio, Gilda De Paola, Giuseppe Sammarco

**Affiliations:** 1 Department of Medical and Surgical Sciences, University of Catanzaro, Viale Europa, 88100 Catanzaro, Italy; vescio@unicz.it (G.V.); gilda_depaola@libero.it (G.D.P.); 2 Department of Health Sciences, University of Catanzaro, Viale Europa, 88100 Catanzaro, Italy; sammarco@unicz.it

**Keywords:** tumor microenvironment, colorectal cancer, targeted therapy, angiogenesis, tumor-associated macrophages, regulatory T cells, cancer-associated fibroblasts

## Abstract

Colorectal cancer (CRC) is a genetically, anatomically, and transcriptionally heterogeneous disease. The prognosis for a CRC patient depends on the stage of the tumor at diagnosis and widely differs accordingly. The tumor microenvironment (TME) in CRC is an important factor affecting targeted cancer therapy. The TME has a dynamic composition including various cell types, such as cancer-associated fibroblasts, tumor-associated macrophages, regulatory T cells, and myeloid-derived suppressor cells, as well as extracellular factors that surround cancer cells and have functional and structural roles under physiological and pathological conditions. Moreover, the TME can limit the efficacy of therapeutic agents through high interstitial pressure, fibrosis, and the degradation of the therapeutic agents by enzymatic activity. For this reason, the TME is a fertile ground for the discovery of new drugs. The aim of this narrative review is to present current knowledge and future perspectives regarding the TME composition based on strategies for patients with CRC.

## 1. Introduction

Colorectal cancer (CRC) is among the three leading causes of cancer-related deaths, with an estimated one million new cases and 600,000 deaths per year globally [1]. The prognosis for a CRC patient depends on the stage of the tumor at diagnosis [2]. The current options for the standard treatment of CRC include surgical removal for stage I and most stage II CRCs and surgical removal and/or adjuvant radiochemotherapy (RCT) for high-risk stage II and stage III CRCs. For metastatic stage IV disease, the surgical removal of the primary CRC and/or metastatic lesions is not always indicated but may be followed by a variety of chemotherapeutic and targeted treatments.

The mortality rate widely differs by stage, being 8–13% for stage I/II, 11–47% for stage III, and almost 90% for stage IV [3]. The last, even after microscopically complete resection (R0), is associated with frequent disease recurrence within five years [4].

However, the recent advent of molecularly targeted therapies, such as cetuximab, panitumumab, and bevacizumab, which can be applied in combination with chemotherapy [5,6], has led to significant increases in progression-free survival (PFS) and overall survival (OS).

**Figure 1:**
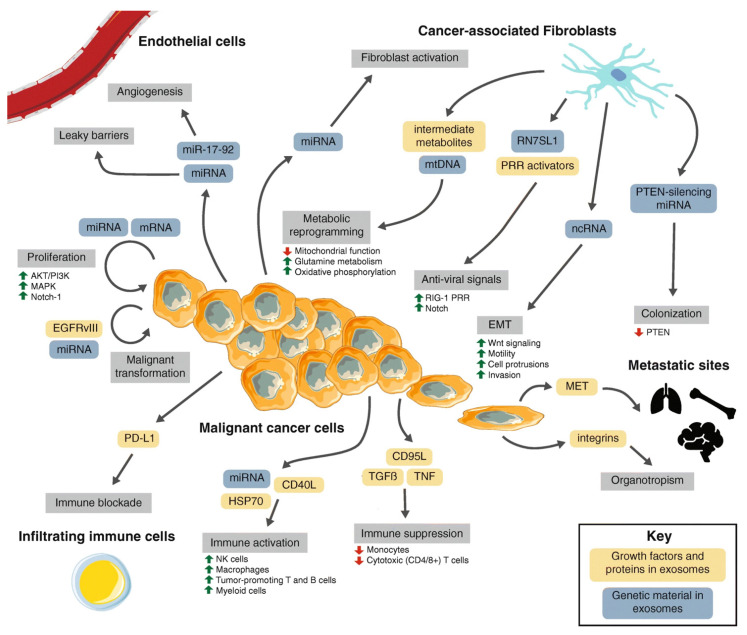
Tumor microenvironment interactions. A macroscopic view of the molecular crosstalk between cancer-associated fibroblasts, endothelial vasculature, infiltrating immune cells, and malignant cells in the TME. Dynamic interactions governed by heterotypic signaling mechanisms between cell types modulate various stages of cancer progression (grey boxes). The role of exosomes in this cell-cell signaling is highlighted (blue and orange boxes). From Li et al. [7].

The tumor microenvironment (TME) has a dynamic composition, including various cell types, such as cancer-associated fibroblasts (CAFs), tumor-associated macrophages (TAMs), regulatory T cells (Tregs), and myeloid-derived suppressor cells (MDSCs), as well as extracellular factors that surround cancer cells (Figure 1) [7] and have functional and structural roles under physiological and pathological conditions.

**Figure 2:**
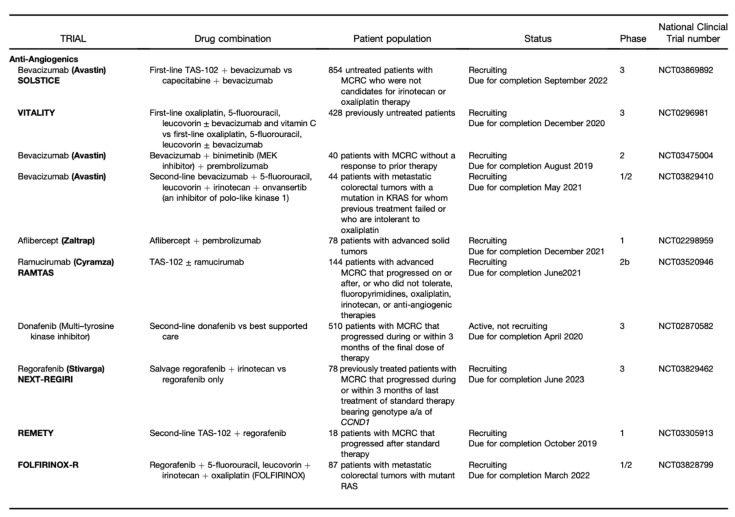
Part I: Clinical Trials of Agents Designed to Target Tumor Stroma. From Fridman et al [9].

**Figure 3:**
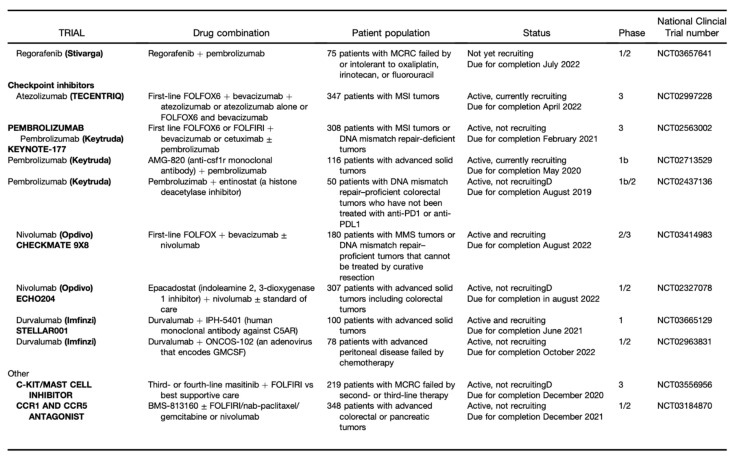
Part II: Clinical Trials of Agents Designed to Target Tumor Stroma. From Fridman et al [9].

The TME can limit the efficacy of therapeutic agents through high interstitial pressure, fibrosis, and the degradation of the therapeutic agent by enzymatic activity. Therefore, it is a fertile ground for the discovery of new drugs (Figure 2 and Figure 3) [8].

It is a special niche in terms of acidity, hypoxia, and ischemia [10], and its components can modulate tumor progression by stimulating angiogenesis, suppressing apoptosis, or inducing immunodepression [11].

In 1971, Judah Folkman first described a revolutionary theory on the role of angiogenesis as a hallmark of cancer. In particular, the “angiogenic switch” is induced by hypoxic tumor cells that stimulate the overproduction of pro-angiogenic factors such as the vascular endothelial growth factor (VEGF) [12].

A better understanding of the TME is essential for the improvement and design of novel therapeutic strategies for colorectal cancer [13,14]. The aim of this narrative review is to present current knowledge regarding the composition of the TME and strategies for patients with CRC.

## 2. Definition

CRC is a genetically (mutation status, i.e., microsatellite instability (MSI)), anatomically, and transcriptionally (different molecular subtypes (CMSs)) heterogeneous disease [13,15].

**Figure 4:**
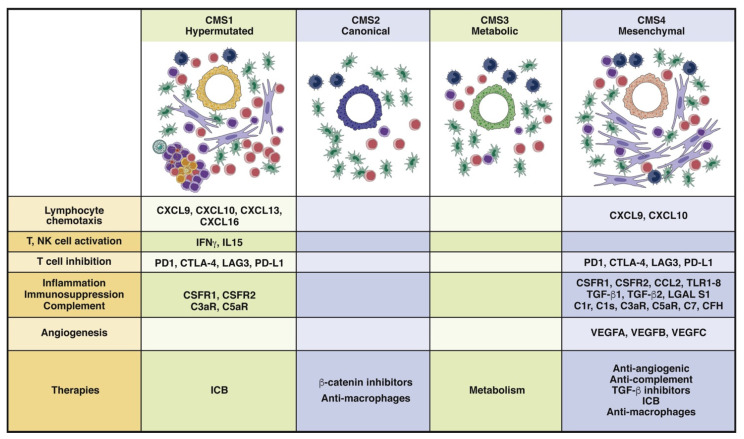
The consensus molecular subtypes. CMS1 and CMS4 tumors are highly infiltrated by immune cells, whereas CMS1 tumors are characterized by a Th1-cell response and activated and inflamed TME. These tumors can be treated with immune checkpoint inhibitors. CMS4 tumors have an inflamed, complement-rich, suppressive, and highly angiogenic TME that can be targeted with combination therapies. CMS2 tumors do not activate an antitumor immune response due to activation of the b-catenin pathway, and CMS3 tumors are considered to be metabolic tumors. CMS1 (14% of colorectal tumors); CMS2 (37% of colorectal tumors); CMS3 tumors (13% of colorectal tumors); CMS4 tumors (23% of colorectal tumors). From Fridman et al. [9].

Concerning the last, a molecular classification system based on six independent datasets for CRC gene expression has been developed for classifying both the tumor and the corresponding TME [16,17]. There are four different CMSs (Figure 4) [9].

CMS1 and CMS4 are subtypes with large proportions of stromal tissue [18] and are associated with the lowest survival rates and worst prognoses for DFS and ODS. Interestingly, CAFs are widely present in the CMS4 subtype [17].

At the beginning of 2000, Hanahan and Weinberg described six essential alterations required in cell physiology to induce malignant growth in almost all tumors [19]. These characteristics can be found in the TME and are as follows: growth factor self-sufficiency, insensitivity to growth-inhibitory signals, the evasion of apoptosis, limitless replicative potential, sustained angiogenesis, and the ability to invade and metastasize.

The idea of the TME or tumor stroma was first introduced at the end of the 19th Century by Paget [20] using the concept of a “seed” (cancer cells) and “soil” (the microenvironment). Paget suggested that the crosstalk between the TME and cancer cells should be closely studied to solve the problems of recurrence, metastasis, and drug resistance [21]. According to this theory, by evaluating preferential patterns of metastatic dissemination to certain organs, it can be observed that the TME provides support for tumor occurrence and progression. In fact, a combination of the colonizing cancer cells’ adaptability and a favorable environment is essential.

## 3. Angiogenesis and Hypoxia

Angiogenesis is a complex process with crucial roles in tumor growth and metastasis and is balanced by pro- and anti-angiogenic factors. Therefore, various steps of the angiogenic process have been targeted.

Regarding CRC, VEGF was discovered as a blood vessel regulator in the 1980s [22,23] and is one of the most studied protagonists of angiogenesis. In fact, bevacizumab, a monoclonal antibody against VEGF, was the first angiogenesis inhibitor approved for the treatment of renal cancer [24] and, subsequently, was used as a first-line option for metastatic CRC (mCRC) following the publication of data obtained by a combined analysis by Kabbinavar and colleagues [25]. They demonstrated, in three clinical trials, that patients with mCRC who were treated with fluorouracil/leucovorin alone or in combination with Bevacizumab showed an increase in mean OS, from 15.6 to 20.3 months, compared to controls.

The survival outcomes of patients with mCRC were further improved after the approval of the antibodies cetuximab and panitumumab, which target the epidermal growth factor receptor (EGFR) [26,27], a crucial element with a key role in the regulation of cell proliferation. In particular, the CRYSTAL trial demonstrated the efficacy of treating patients with wild-type KRAS with cetuximab in combination with folinic acid, 5-fluoruracil, and irinotecan (FOLFIRI) or leucovorin, 5-fluoruracil, and oxaliplatin (FOLFOX) in terms of OS. There was no statistically significant benefit of combination therapy for patients with a KRAS mutation, a well-known predictor of EGFR-therapy resistance [28].

A recent randomized phase III trial conducted in Germany, FIRE-3, demonstrated that FOLFIRI–cetuximab was slightly better than FOLFIRI–bevacizumab in mCRC patients regarding the overall response rate (72% vs. 56.1%; *p* = 0.0029) and OS (33.1 vs. 25.0 months; *p* = 0.0059) [29].

Ramucirumab and aflibercept have been approved, in combination with FOLFIRI, as second-line treatments [30,31]. Aflibercept is a recombinant fusion protein composed of the constant Fc domain of human IgG and the second and third immunoglobulin domains of VEGFR-1 and VEGFR-2, respectively. It acts as a ligand and inhibits the activity of VEGF-A and VEGF-B, rather than that of the placental growth factor (PIGF) [32]. Interestingly, no significant improvement in survival outcomes was demonstrated when aflibercept was used after bevacizumab [31]. Ramucirumab is a fully human monoclonal antibody (IgG1) that normally blocks the interaction between VEGF and its receptor and has already produced satisfactory results in treating lung and gastric cancers [33,34] but not mCRC.

Lastly, the angiopoietins (Ang-1; Ang-2; Ang-3; Ang-4) and their receptor Tie2 (TEK) deserve to be considered for the excellent results shown in preclinical models [35,36]. The search for new anti-angiogenic strategies continues, and trebananib and many others are under evaluation.

There is an important connection between angiogenesis and hypoxia. A hypoxic TME is characteristic of most locally advanced solid tumors and contributes to therapy resistance [37,38]. Tumorigenesis is associated with high oxygen requirements that often cannot be satisfied by the surrounding cells, resulting in hypoxia. VEGF gene expression is stimulated by the latter, and the whole process is coordinated by the transcriptional factor hypoxia-induced factor-1 (HIF-1). Accordingly, topotecan, a topoisomerase 1 inhibitor, has been used for the treatment of solid tumors expressing HIF-1α [39,40]. Additionally, an antisense oligodeoxynucleotide targeting HIF-1α, EZN-2968, was recently developed [41] but still has a long way to go before becoming an approved therapy.

## 4. Cancer-Associated Fibroblasts

CAFs are the predominant non-malignant tumor cells in the TME and have an origin that is variable and not completely understood [42,43]. They may arise from epithelial cells, human bone marrow-derived mesenchymal cells, adipocytes, or hematopoietic stem cells in response to a stimulus mediated by EMT. Another source of CAFs is normal fibroblasts that undergo genetic alterations involving the inactivation of phosphatidylinositol-3, 4, 5-trisphosphate 3-phosphatase (PTEN) and p53 [44].

Fibroblasts can initially block the evolution of early-stage tumors [45]. However, they can later be modulated by tumor cells and stimulated to transform into CAFs. Transforming growth factor β (TGF-β) is the main chemotactic agent for fibroblasts that drives transdifferentiation into CAFs [46]. Moreover, TGF-β has a pro-tumorigenetic effect on neutrophils and macrophages [47]. Unlike the other components of the TME, the transformation of fibroblasts is due to a chronic stimulus mediated by tumor cells, which can be defined as epigenetic [48] and is perfectly in line with the previously described two-step activation model (reversible–irreversible) [49].

Once they are activated, their presence in the TME is associated with poor clinical outcomes [45,50] due to the secretion of several molecules, such as stromal cell-derived factor 1 (SDF-1), which stimulates tumor angiogenesis [51] (through VEGFB, VEGFC, and PDGFC) and cancer cell colonization and metastasis [52].

Currently, therapy targeted at CAFs is mainly aimed at reducing oxidative stress, a mediator of the metabolic symbiosis between CAFs and tumor cells, and TGF-β [53,54,55]. Regarding the latter, several results have already been obtained [56,57].

## 5. Regulatory T Cells

Regulatory T cells (Tregs) are a subgroup of T-cells that, under physiological conditions, have several immunomodulatory effects on B and T cells, such as preserving the homeostasis of cytotoxic lymphocytes [58]. However, depending on the environmental stimuli received, Tregs can promote tumorigenesis. In particular, Tregs may suppress the immune response to autologous tumor cells, rendering immunotherapy ineffective [59,60].

**Figure 5:**
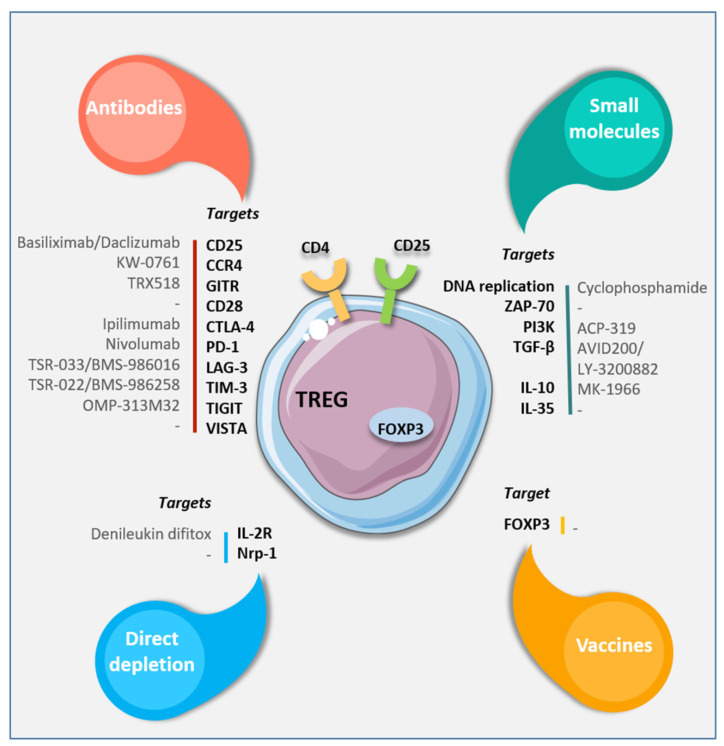
Targeting strategies to eliminate or modulate Treg functions. Available antibodies, small molecules, or vaccines specific for different cell surface or intracellular targets. From Laplagne et al. [61].

A variety of targets for suppressing Tregs’ activity have recently been identified (Figure 5) [61]. 

The transcription factor forkhead box P3 (Foxp3) is one of the most specific Treg markers and, consequently, a target for new therapeutic strategies. Moreover, due to the expression of the cytotoxic T lymphocyte-associated antigen-4 (CTLA-4), which interacts with B7, it has tumorigenetic activity (inhibits T-cells) [62].

Daclizumab is an FDA-approved monoclonal antibody raised against the CD25 receptor that has been proven to be effective for decreasing circulating T-regs, reducing the expression of both CD25 and Foxp3 [63], and increasing the release of IFN-y.

Other than CTLA-4 and CD-25, further effort is currently aimed at blocking the activity of CD-28, one of the main stimuli for Treg activity [64].

## 6. Tumor-Associated Macrophages

TAMs are key immune cells that are present in high concentrations in the TME [65,66]. Once recruited by growth factors such as colony-stimulating factor-1 (CSF-1) and VEGF, inflammatory monocytes that have migrated into the TME differentiate into TAMs and produce immunosuppressive cytokines such as PGE2 and TGF-β for the suppression of T-cell proliferation [67].

They are highly plastic [68,69] and act as either anti- or pro-tumor agents depending on environmental stimuli, causing polarization into two phenotypes, classically activated macrophages (M1 cells) and alternatively activated macrophages (M2 cells) [65], which have roles in adaptive immunity corresponding to Th1 and Th2, respectively.

Their accumulation is often associated with a poor prognosis and enhanced metastasis in most solid cancers. In particular, a low M1/M2 ratio is related to a carcinogenic pattern [70], considering that M1 cells are usually activated by IFN-I, which, along with Notch signaling and IFN-I, has an anti-tumor effect [70,71,72].

Conversely, M2 macrophages are involved in angiogenesis, tissue modeling and repair, and the differentiation of regulatory T-cells [73] and are driven by M-CSF, interleukin-3 (IL-3), IL-4, and IL-13 [74]. Furthermore, they participate in inflammation-associated carcinogenesis.

In a recent meta-analysis including 55 studies and 8692 patients, Zhang et al. [75] evaluated the infiltration of TAMs in patients with solid tumors using a pan-macrophage marker: CD68.

Interestingly, a high density of TAMs was associated with positive survival outcomes, only in patients with CRC. Nevertheless, colonic carcinoma may induce TAMs to promote angiogenesis and metastasis, contributing to the first step of cancerogenesis, that is, the epithelial-to-mesenchymal transition (EMT) [76,77].

Colon carcinoma cells are known to produce CSF-1 [76,78,79], which recruits macrophages to the tumor periphery, where they secrete promotility and angiogenic factors that facilitate tumor cell invasion and metastasis.

**Figure 6:**
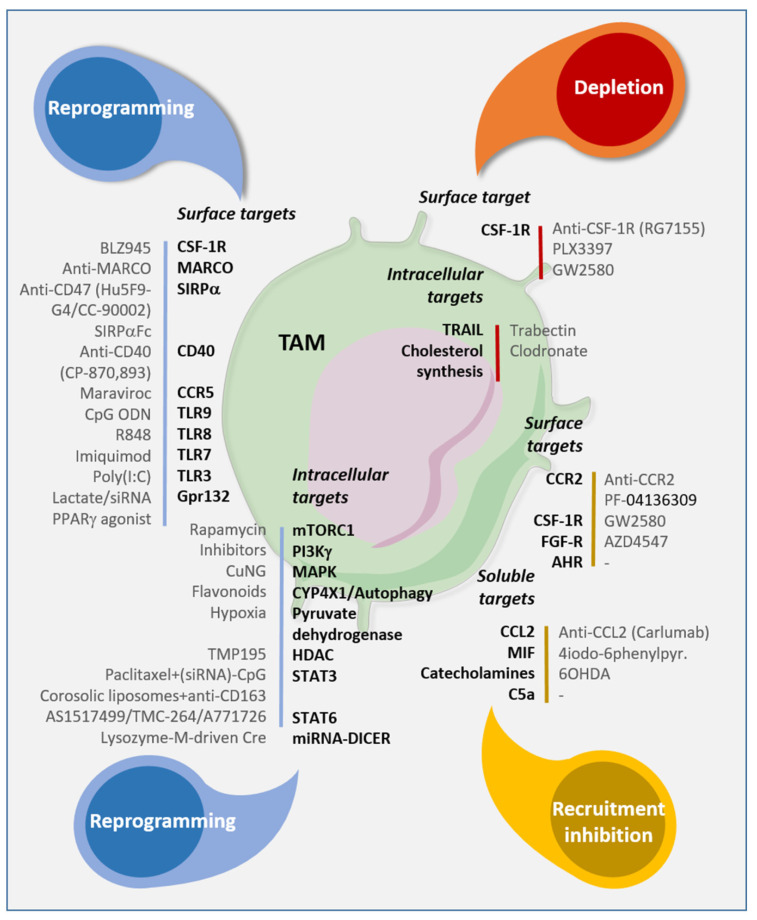
Targeting strategies to reprogram, eliminate, and inhibit TAM recruitment. Antibodies or molecules available to target surface, intracellular or soluble molecules involved in the phenotype, functions, and recruitment in the TME. From Laplagne et al. [61].

TAMs modulate the ECM. Matrix metalloproteinases, which are ECM-remodeling enzymes, regulate signaling pathways that control cell growth, inflammation, and angiogenesis. TAMs are a current target for immunotherapy. In fact, reprogramming towards an M1 phenotype could increase the effectiveness of chemotherapeutically targeted therapies (Figure 6) [80].

## 7. Myeloid-Derived Suppressor Cells

MDSCs represent a population of granulocytes and monocytes which, together with tumor-associated neutrophils (TANs), tumor-associated macrophages (TAMs), and regulatory dendritic cells, constitute the population of myeloid regulatory cells (MRC) [81,82,83]. This cell population expands rapidly as a result of infectious, inflammatory, and, especially, cancer processes, favoring its development and progression [83].

They were previously defined as ”immature myeloid cells” or “myeloid suppressor cells” (MSC) [84,85], but, as these terms were too generic and misleading, they were replaced in 2007 with MDSCs, in order to better clarify the origin and function of these cells [86].

**Figure 7:**
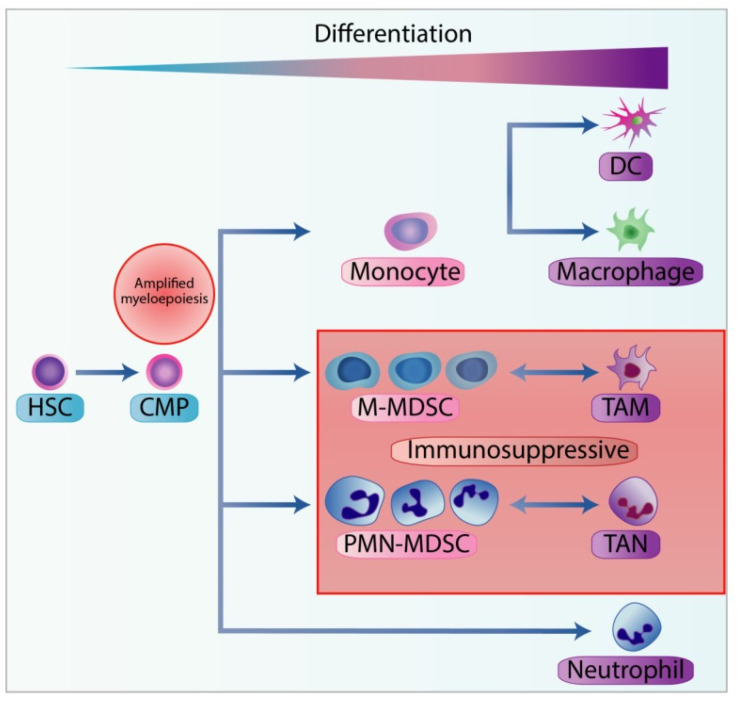
Stages of myelopoiesis differentiation in cancer. Myelopoiesis is amplified during chronic inflammation to assist tumour progression and dissemination. The hematopoietic stem cells (HSC) differentiate into the common myeloid progenitor (CMP), which can further differentiate through the hematopoietic system. In physiological conditions, CMP can differentiate into neutrophils or into monocytes, and subsequently into dendritic cells (DC) or macrophages. However, with chronic inflammation, pro-inflammatory cytokines can skew the monocytopoiesis of CMP into monocytic-myeloid-derived suppressor cells (M-MDSC) and tumour-associated macrophages (TAM), and granulopoiesis into polymorphonuclear myeloid-derived suppressor cells (PMN-MDSC) and tumour-associated neutrophils (TAN). From Law et al [87].

MDSCs can be divided into two groups: granulocytic or polymorphonuclear, phenotypically similar to neutrophils (PMN-MDSC) and monocytic MDSCs (M-MDSC) (Figure 7) [87,88].

Furthermore, the existence of a third type of MDSC, called early-stage MDSCs, which have the ability to form colonies and other myeloid precursors, has been recently demonstrated [89].

The recruitment of MDSCs is mediated by various factors released during chronic inflammation such as chemokines [82,88,90,91], histamine [92,93] and prostaglandin E2 [94].

Among the chemokines, the C–C motif chemokine Ligand 2 (CCL2) plays a fundamental role in both determining the accumulation of MDSCs and increasing their immunosuppressive action with the consequent growth, progression, and development of metastases in patients with CRC [95].

Histamine can lead to the recruitment of Mo-MDSCs and PMN-MDSCs in different ways [92,93]. First, it promotes the expression of the enzyme arginase-1 (ARGI1) and inducible NO synthase (iNOS) in Mo-MDSCs; second, it inhibits ARGI1 and iNOS in PMNMDSCs by inducing the production of IL-13 and IL-14 [92,93]. The latter enzymes are fundamental in the correct metabolism of L-arginine and are involved in the proliferation of T lymphocytes, the expression of the CD3ζ chain, and the production of IFNγ [96,97,98,99].

Prostaglandin E2 (PGE2) is another crucial proinflammatory factor produced by COX-2 that determines the recruitment of MDSCs following STAT3 (Signal Transducer and Activator of Transcription 3) phosphorylation [100,101]. Consequently, persistent activation of the STAT3 pathway is associated with the growth of CRC cells [102,103]. Interestingly, the role of MDSCs was also observed in precancerous lesions [104].

Furthermore, MDSCs also play a role in determining high levels of MMP9 and pro-MMP9, which result in extracellular matrix degradation; they increase VEGF and, in the context of metastases, favor the transition process from epithelial to mesenchymal cells (EMT) [105,106,107,108].

There is, therefore, clear evidence of the involvement of MDSCs in the colorectal carcinogenesis process, and this could justify the use of these cells in the determination of prognosis and as therapeutic targets [83].

**Figure 8:**
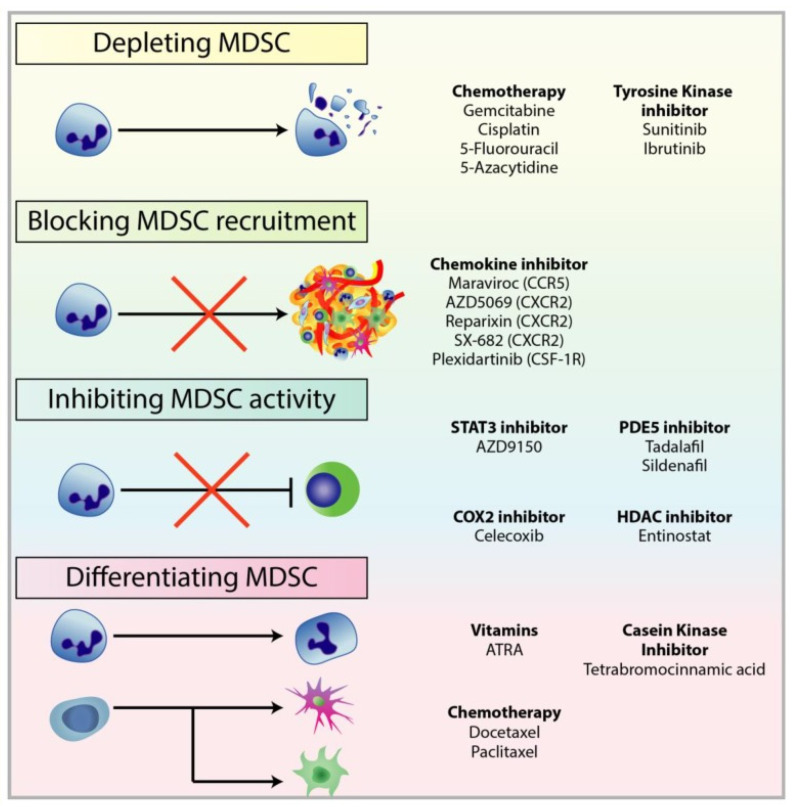
Treatments used to target different mechanisms associated with pro-tumourigenic MDSC. There are multiple therapeutic approaches against MDSC to restore anti-tumour functions in immune cells and improve immunotherapy, in particular checkpoint inhibitors. These approaches include: (1) depleting MDSC populations through low-dose chemotherapy and tyrosine kinase inhibitors; (2) preventing MDSC recruitment to the TME by targeting chemokine receptors responsible for the recruitment and migration of MDSCs; (3) attenuating the immunosuppressive mechanisms of MDSC by downregulating the expression of ARG1 and iNOS, and reducing ROS generation; (4) inducing the differentiation of MDSC into mature myeloid cells to reduce MDSC population and remove their immunosuppression. From Law et al [87].

To date, there are several therapeutic approaches to combat the immunosuppressive action of MDSCs (Figure 8) [87]: (1) the first approach consists of reducing the number of MDSCs previously recruited into the neoplastic process through low-dose chemotherapy with 5-fluorouracil (5FU), paclitaxel, cisplatin or gemcitabine [109,110,111,112] and through tyrosine kinase inhibitors, such as Sunitinib [113,114]; (2) the second mechanism acts upstream, targeting the chemokine receptors involved in the recruitment of MDSCs [115,116,117]; (3) the third determines the down-regulation of ARG1 and iNOS, which act on the metabolism of L-Arginine [118,119]; (4) the fourth approach aims to promote the differentiation of MDSCs in mature myeloid cells with Tretinoin, also known as all-trans retinoic acid (ATRA) [120], in order to reduce their immunosuppressive effect.

However, targeting MDSCs in monotherapy, as well as using immunotherapy alone, can sometimes lead to lead to insufficient outcomes in cancer treatment. For this reason, there are studies supporting the use of combination therapies [117,121,122].

## 8. Emerging Treatments and Others

Immunotherapies using nanoparticles have recently drawn attention due to the possibility of both increasing drug delivery into solid tumors and avoiding the problem of drug resistance [123].

Organic and inorganic nanoparticles have been developed and can be used as carriers of antigens, proteins, or therapeutic agents, promoting specific and effective immune responses [124,125].

In fact, the delivery of chemo-agents may induce immunogenic cell death [126] in the TME and activate tumor-infiltrating antigen-presenting cells (APCs) [127]. Furthermore, nanoparticles can modulate a hypoxic TME by increasing oxygen release. Interestingly, Song et al. demonstrated the effectiveness of combined oxaliplatin-programmed death ligand-1 inhibition therapy in a colorectal cancer murine model [128]. Even though there are several delivery methods for nanoparticles, such as exosomes, chitosan, plasma membrane coating, and mesenchymal stem cells, there is still no standardization, especially regarding their efficacy and safety [123].

Two other important components of the TME are matrix metalloproteinases (MMPs) and mast cells.

MMPs are zinc-containing, calcium-dependent endopeptidases. They are responsible for the degradation of the extracellular matrix (ECM) and, consequently, tissue remodeling [129]. Their physiological expression is regulated by multiple hormones, growth factors, and cytokines, but their overexpression is involved in several disorders such as hemorrhoidal disease [130], inflammatory bowel disease [131], and cancer [132]. They are often expressed in advanced CRC and are associated with poor survival outcomes [133].

Mast cells are innate immune cells with a crucial role in the TME, and they represent the most studied components of the latter. A high mast cell density is correlated with increased vascularity, enhanced tumor growth, invasion, and poor clinical outcomes [134]. A low number of mast cells is associated with better survival in CRC [135]. Once activated, mast cells act as a trigger for the angiogenic switch, stimulating the production of VEGF, histamine, TNF-α, and several proteases [13]. The use of mast cell and MMP inhibitors [136] as adjuvant or neoadjuvant therapies in solid tumors [137] is currently under evaluation.

Our study has some limitations. This was a comprehensive narrative review that considered the main components of the TME. As a result, some elements that may play a role in the future, but are currently under development, may have been overlooked.

## 9. Conclusions

The tumor microenvironment is an important factor that affects targeted therapies for colorectal cancer. The downregulation of the pathways activated by the components most frequently present in the TME may improve the prognosis associated with the disease. However, future studies confirming this potential are needed.

## Data Availability

Not applicable.
